# The Shank3^Venus/Venus^ knock in mouse enables isoform-specific functional studies of Shank3a

**DOI:** 10.3389/fnins.2022.1081010

**Published:** 2022-12-08

**Authors:** Nathalie Bouquier, Sophie Sakkaki, Fabrice Raynaud, Anne-Laure Hemonnot-Girard, Vincent Seube, Vincent Compan, Federica Bertaso, Julie Perroy, Enora Moutin

**Affiliations:** ^1^Institut de Génomique Fonctionnelle, Université de Montpellier, CNRS, INSERM, Montpellier, France; ^2^PhyMedExp, Univ Montpellier, INSERM, CNRS, CHU de Montpellier, Montpellier, France

**Keywords:** Shank3, knock in, ASD model, isoform-specific tagging, Shank3Δ⁢C, Shank3a

## Abstract

**Background:**

Shank3 is a scaffolding protein essential for the organization and function of the glutamatergic postsynapse. Monogenic mutations in *SHANK3* gene are among the leading genetic causes of Autism Spectrum Disorders (ASD). The multiplicity of Shank3 isoforms seems to generate as much functional diversity and yet, there are no tools to study endogenous Shank3 proteins in an isoform-specific manner.

**Methods:**

In this study, we created a novel transgenic mouse line, the Shank3^Venus/Venus^ knock in mouse, which allows to monitor the endogenous expression of the major Shank3 isoform in the brain, the full-length Shank3a isoform.

**Results:**

We show that the endogenous Venus-Shank3a protein is localized in spines and is mainly expressed in the striatum, hippocampus and cortex of the developing and adult brain. We show that Shank3^Venus/+^ and Shank3^Venus/Venus^ mice have no behavioral deficiency. We further crossed Shank3^Venus/Venus^ mice with Shank3^ΔC/ΔC^ mice, a model of ASD, to track the Venus-tagged wild-type copy of Shank3a in physiological (Shank3^Venus/+^) and pathological (Shank3^Venus/ΔC^) conditions. We report a developmental delay in brain expression of the Venus-Shank3a isoform in Shank3^Venus/ΔC^ mice, compared to Shank3^Venus/+^ control mice.

**Conclusion:**

Altogether, our results show that the Shank3^Venus/Venus^ mouse line is a powerful tool to study endogenous Shank3a expression, in physiological conditions and in ASD.

## Introduction

Autism Spectrum Disorders (ASDs) affect about 1% of the human population. Patients with ASD have impairments in social communication and interaction and present repetitive and stereotyped behaviors (American Psychiatric Association, 2013). A significant proportion of ASD is due to genetic mutations, which has given hope for treatment (Kleijer et al., 2014). The discovery of monogenic forms of ASD offers a unique opportunity to explore the molecular and cellular mechanisms underlying ASD. The striking convergence of “ASD genes” onto glutamatergic signaling (Connor et al., 2014; Lee et al., 2016; Brown et al., 2018) supports the synaptic hypothesis as a leading cause of autism (Bourgeron, 2015), with the prominent role played by postsynaptic proteins linking group I mGlu receptors with AMPA and NMDA receptors (Connor et al., 2014). Heterozygous mutations in the *SHANK3* gene are among the leading monogenic causes of ASD and can lead to Phelan McDermid syndrome (PMS) (Durand et al., 2007; Jiang and Ehlers, 2013; Sala et al., 2015; Monteiro and Feng, 2017). PMS is characterized by a significant delay in expressive speech, intellectual disability, hypotonia, dysmorphic facial features, increased tolerance to pain, epilepsy, and autism-like behavior (Costales and Kolevzon, 2015).

**FIGURE 1:**
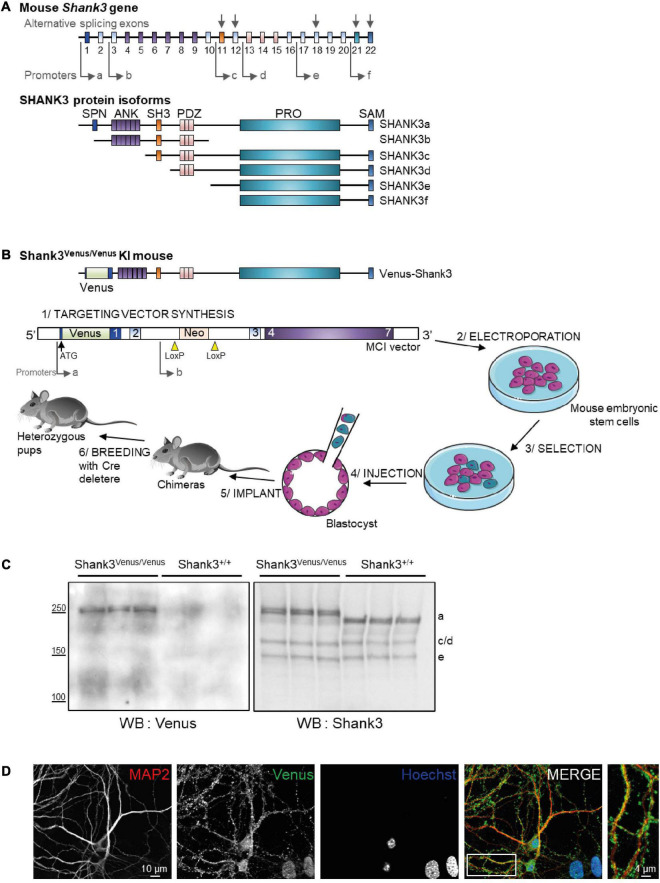
Generation of the Shank3^Venus/Venus^ knock in mouse line and detection of Venus Shank3a isoform expression. **(A)** Schematic representation of the mouse Shank3 gene structure and protein domains of the different protein isoforms. Sites of intragenic promoters and alternative splicing exons are represented by arrows. SPN for Shank3/ProSAP N-terminal domain (1-75 aa); ANK for Ankyrin repeat domain (148-345 aa); SH3 for src Homology-3 domain (470-529 aa); PDZ for PSD-95/DLG/ZO-1 domain (570-664 aa); PRO for proline-rich domain (831-1417 aa); SAM (1667-1730 aa). **(B)** Schematic representation of the Venus-Shank3 protein domains and steps of Shank3^Venus/Venus^ KI mouse line creation. Please note that the Venus coding sequence was inserted at the beginning of exon 1 (in frame with and just after the ATG), enabling the exclusive Venus-tagging of the Shank3 variants that are generated by the first promoter, Shank3a isoforms. **(C)** Total brain lysates from 3 different Shank3^Venus/Venus^ and Shank3^+/+^ adult mice were immunoblotted for Venus (left) and Shank3 (right) expressions. Expected molecular weight for untagged Shank3a, 3c/d and 3e isoforms are indicated on the right side of the blot (a, c/d, and e). **(D)** Immunocytochemistry images from *Day In Vitro* 15 Shank3^Venus/Venus^ hippocampal primary cultures stained with antibodies against the dendritic marker MAP2 (red), Venus (green), and the nuclear marker Hoechst (blue).

The *SHANK3* gene is located on mouse chromosome 15E3 and human chromosome 22q13.3. There are at least 6 different Shank3 protein isoforms, Shank3a to Shank3f (Figure 1A), the full-length isoform (Shank3a) being the most abundant form in the brain (Wang et al., 2014). Shank3a is also the longest isoform and includes a Shank/ProSAP N-terminal domain (SPN), 6 ankyrin repeats (ANK), a Src Homology 3 domain (SH3), a PDZ (PSD-95/Dlg1/ZO-1) domain, a proline-rich region (Pro-rich), and a sterile alpha motif (SAM) domain at the C-terminus (Naisbitt et al., 1999; Tu et al., 1999). These protein-protein interaction domains multiply the possibility of interactions of Shank3a protein with dozens of synaptic proteins, making it a key component of the glutamatergic post-synaptic architecture. Unfortunately, due to the lack of specific antibodies or genetic tools, there is currently no possibility to study this Shank3a isoform exclusively.

In an attempt to study the physiological role of endogenous Shank3a, we generated a Shank3^Venus/Venus^ knock in (KI) mouse. This KI strategy is adding a tag on the protein of interest while bypassing the need to overexpress the coding gene, thus providing information on the endogenous protein at physiological expression levels in an intact cellular context. In addition, only the major isoform, Shank3a, is tagged in this mouse line, allowing to focus on Shank3a expression and function amongst all other isoforms. Finally, this new mouse line can be crossed with Shank3 mutant mice to study the repercussion of such mutations on Venus-Shank3a wild type protein in heterozygous mouse models of ASD. This strategy, focusing on the physiological role of endogenous Shank3, complements the previous attempts to understand how alterations of the Shank3 protein can lead to the development of ASD (Yoo et al., 2013; Sala et al., 2015; Varghese et al., 2017).

## Materials and methods

### Generation of *Shank3^Venus/Venus^* KI mice at the Mouse Clinical Institute and genotyping

The targeting vector was constructed as follows. A 3.6 kb 3′ homology arm fragment encompassing exons 3–7 was amplified by PCR and subcloned in an Mouse Clinical Institute (MCI) proprietary vector. This vector bares a floxed Neomycin resistance cassette associated with a Cre auto-excision transgene that allows the excision of the whole cassette in the chimera’s male germ line. A 1.5 kb fragment corresponding to the fusion of two PCRs amplicons (the Venus tag sequence and a 740-bps genomic fragment encompassing exon 2) was cloned in a second step using the endogenous SrfI site in 5′ and finally, a 3.6 kb (GC rich as covering the 5′ UTR of the gene) fragment corresponding to the 5′ homology arms was amplified by PCR and subcloned in step 2 plasmid to generate the final targeting construct. The linearized construct was electroporated in C57BL/6N mouse embryonic stem (ES) cells. After selection, targeted clones were identified by PCR using external primers and further confirmed by Southern blot with a Neo probe (5′ and 3′ digests) as well as a 3′ external probe. Three positive ES clones were injected into BALB/cN blastocysts. One gave germ line transmission. Resulting male chimeras were bred with wildtype C57BL/6N females. Germline transmission of the knock in allele with a direct excision of the floxed selection cassette was obtained. The allele nomenclature (according to Mouse Genome Informatics) is Shank3^tm1(Venus)Ics^.

The primers used for genotyping have the following sequence: GGTACGGCGAGATCGCAAAGG and CTCTCTCC GCCGGGAACAG. The size of the PCR products is 862 bp for the KI allele and 133 bp for the WT allele.

### Animal handling

All animal procedures were conducted in accordance with the European Communities Council Directive, supervised by the IGF institute’s local Animal Welfare Unit (A34-172-41) and approved by the French Ministry of Research (agreement numbers: APAFIS#23357-2019112715218160 v4 and APAFIS#23476-2020010613546503 v4). The Shank3^ΔC/ΔC^ mice (Jackson Laboratory, Bar Harbor, ME, USA, stock #018398) have a deletion of the Shank3 3′ terminal part, starting just before exon 21. The resulting protein is truncated before the Homer-binding domain. Homozygous Shank3^Venus/Venus^ mice were bred with homozygous Shank3^ΔC/ΔC^ to obtain Shank3^Venus/ΔC^ mice (Figure 5A). Heterozygous Shank3^Venus/+^ mice were crossed together to obtain Shank3^Venus/Venus^ mice, Shank3^Venus/+^ mice and Shank3^+/+^ mice. Mice were grouped after weaning with respect to their sex.

### Western blot analysis

Mice of both sexes were anesthetized with isoflurane. Brain structures were solubilized in a 10% SDS solution supplemented with protease inhibitors (Roche Diagnostics, Germany). Samples were sonicated and centrifuged. Concentration of solubilized proteins was quantified using BCA assay (Sigma-Aldrich, USA). All samples were then set at the same volume and concentration for the rest of the protocol and 30ug of proteins were loaded for each condition. Proteins were eluted in Laemmli sample buffer, resolved on a tris-acetate 3-8% gradient gel (BioRad, USA) enabling the detection of all expected bands at different molecular weights into the same gel (from Venus-Shank3a to GAPDH). Proteins were transferred onto a nitrocellulose membrane, which was cut in two pieces upper and lower than 75 kDa and then incubated with a mouse anti-GFP antibody and anti-GAPDH antibody for the upper and lower part, respectively. After this first immunostaining, we applied 0.5% azide-containing solution to the upper part of the membrane to quench the chemiluminescence and incubated the rabbit anti-Shank3 antibody for a second revelation of this upper part of the gel. Immunoblot detection was performed using the following primary antibodies: GFP (mouse monoclonal from Clontech, USA, reference Cat.#632381, dilution 1:500), GAPDH (rabbit polyclonal from Santa Cruz reference, dilution 1:25,000), Shank3 (rabbit polyclonal from Santa Cruz, USA, reference SC-30193, dilution 1:500). A Mann–Whitney test was used for experiments comparing 2 conditions and a non-parametric Kruskal–Wallis test with uncorrected Dunn’s post-test for those comparing more than 2 conditions.

### Hippocampal primary cell culture

Cultures were prepared from postnatal Shank3^Venus/Venus^ mice as previously described (Moutin et al., 2020). Briefly, hippocampi were mechanically and enzymatically dissociated with papain (Sigma-Aldrich, USA) and hippocampal cells were seeded in Neurobasal-A medium (Gibco, USA) supplemented with B-27, Glutamax, L-glutamine, antibiotics and Fetal Bovine Serum (all media from Gibco, ThermoFisher Scientific, USA). After 2 days in culture, cytosine β-D-arabinofuranoside hydrochloride (Sigma-Aldrich, USA) was added to curb glia proliferation. The day after, 75% of the medium was replaced by BrainPhys medium (Stemcell Technologies, Canada) supplemented with B-27, Glutamax and antibiotics.

### Immunocytochemistry

Shank3^Venus/Venus^ hippocampal primary cultures were fixed at *Day In Vitro* 15 with 4% paraformaldehyde for 10 min and then permeabilized and blocked with a 3% BSA, 0.1% Triton X-100, PBS solution (blocking buffer) for 1 h at room temperature. Cultures were then incubated overnight at 4°C with primary antibodies against GFP (TP401, Biolabs, USA) to stain Venus, and against MAP2 (M4403, Sigma-Aldrich, USA) diluted in blocking buffer to final concentrations of 1:1,000. After washes, cells were incubated with secondary antibodies for 2 h at room temperature, washed, incubated with Hoechst 33258 (B2883, Sigma-Aldrich, USA) diluted in water for 5 min at room temperature, mounted on slides and observed under an Axio-Imager Z1 microscope equipped with appropriate epifluorescence and filters (Carl Zeiss, Germany).

### Behavioral experiments

In order to characterize the psychomotor development of Shank3^Venus/Venus^ mice, weight, flipping, cliff avoidance and walking tests were performed each day from P0 to P15 on both male and female pups. Self-grooming, Three chambers sociability test, Open field and Marble burying were performed on male and female mice aged 12–14 weeks.

*Weight*: The mice were weighed each day on a precision balance.

*Flipping*: Mice were placed on their backs. Those remaining on their backs for at least one minute were scored 0. Those that flipped onto their stomachs within 30 s to 1 min, 15–30 s, or 5–15 s were scored 1, 2, 3, respectively. Mice that flipped over in less than 5 s received a score of 4.

*Cliff avoidance*: Mice were placed on the edge of a Plexiglas platform over a 20 cm cliff with their nose and front legs over the edge. Mice that moved away from the edge within 30 s received the maximal score of 1, while mice that did not move got the score of 0.

*Walking*: Mice were observed for 1 min. Mice that remained immobile or moved in circles received a score of 0. Mice that walked but inconsistently and asymmetrically received a score of 1. Mice that walked symmetrically but slowly got a score of 2 while those that walked correctly and quickly received a maximum score of 3.

*Open Field* consisted of a square arena (50 cm × 50 cm). Mice were placed in the center of the arena and left to explore freely for 10 min. Total distance traveled, speed and time spend in the center zone (defined as a 25 cm side square) were measured during 10 min by video tracking (Ethovision, Noldus, Netherlands).

*Self-grooming*: Mice were placed in a 20 cm Plexiglas square arena and video monitored. Time spend self-grooming was measured visually during 10 min.

*Marble test*: Mice were placed individually in a clean cage with 5 cm thick bedding together with 15 marbles evenly distributed on top of the bedding. After 30 min in the cage, mice were removed and we evaluated the level of marble burying according to the following score: 0 for a totally buried marble; 1 for a half-buried marble and 2 for a totally visible marble. We also evaluated the level of interaction with the marbles counting marbles that were untouched during the test.

*Three chambers sociability test*: Mice were tested individually in a three chambers test apparatus (rectangular 40 × 60 plexiglass, Panlab, Spain). The apparatus was cleaned with alcohol 30% between trials. Each of the two side chambers contained a cage (9 cm in diameter) in which social (stranger mouse) and non-social (object) stimuli could be confined. Testing consisted of three trials: trial 1 (habituation), the test mouse was placed in the middle chamber and then could freely explore the test box for 10 min with the cages empty. After which they returned to their home cage for 1 h before proceeding with the next trial. During trial 2 (social preference), an unknown mouse (stranger 1: social stimulus) was introduced in one of the cages in one side chamber, whereas an unknown object (object: non-social stimulus) was enclosed in the other cage in the opposite side chamber. Location of stranger 1 in the left or right-side chamber was balanced across subjects. The test mice were allowed to explore during 10 min. After 1 h in their home cage, mice were exposed to trial 3. During trial 3 (social novelty), the object present in trial 2 was replaced by a novel unfamiliar mouse (stranger 2: novel social stimulus). Again, the test mouse was allowed to explore during 10min. Behavior was video monitored during all trials (Ethovision, Noldus, Netherlands) to analyze general activity (distance traveled, speed). Social preference index was calculated as the ratio of time spent in the side chamber containing the social stimulus by the time spent in both side chamber during trial 2. Social novelty recognition index was calculated as the ratio of time spent in the side chamber containing the novel social stimulus by the time spent in both side chamber during trial 2.

*Statistical analysis*: For pups, we analyzed the evolution of mice proportion over time using Generalized Linear Model (GLM) (Ordinal Logit model, SPSS^®^ IBM^®^) with score as dependent variable and genotype and days as covariates. We also used GLM to compare the evolution of weight over time with weight as dependent variable and genotype and days as covariate (Linear Scale Response model, SPSS^®^ IBM^®^).

For the adults’ tests, differences between the three genotypes groups were analyzed using One-way ANOVA or Kruskal–Wallis accordingly to their distribution (Graph Pad Prism 9.3.0).

## Results

### Generation of a *Shank3*^Venus/Venus^ KI mouse line allowing the exclusive detection of Shank3a isoform

In mice, the *SHANK3* gene is composed of 22 exons (Figure 1A). Multiple intragenic promoters, at least six (see Monteiro and Feng, 2017 for review), lead to six major protein isoforms: Shank3a to Shank3f. We have generated the Shank3^Venus/Venus^ KI mouse line by inserting the DNA coding for Venus fluorescent tag at the beginning of exon1 of *SHANK3* gene, just after the ATG start codon (Figure 1B). This strategy enables to tag exclusively the endogenous Shank3 variants that are produced by the first promoter: variants of the Shank3a isoform only, Shank3b to f being generated by promotors located downstream Venus DNA coding sequence. To check for the effective expression of this tagged version of Shank3, we performed Western blots on brain samples of adult Shank3^Venus/Venus^ mice with two different primary antibodies. The first one was raised against Venus and the second one against the C-terminal part of Shank3 (Verpelli et al., 2011) (which recognizes Shank3a, c/d e, and f isoforms). Consistently, Western blot analysis of adult Shank3^Venus/Venus^ mouse brains showed only one double band (Figure 1C), with an apparent molecular weight corresponding to the expected size of full-length Venus-Shank3a (theoretical Venus-Shank3a molecular weight: 212 kDa). As expected, the shorter isoforms lacking at least the N-terminal exon 1 and exon2 (Shank 3b to f) could not be detected using an anti-Venus antibody. These shorter bands are present when using the anti-Shank3 antibody labeling all Shank3 isoforms conserving the C-terminal part, excepted for Shank3f which molecular weight is around 10 kDa (Monteiro and Feng, 2017) and cannot be detected in our experimental conditions (Figure 1C). Furthermore, as already reported for Shank3c, Shank3d, and Shank3e, additional transcripts may be produced by alternative splicing of exons (exon 11, exon 12, exon 18, exon 21, and exon 22). For example, for Shank3c, alternative splicing gives rise to Shank3c1, Shank3c3, and Shank3c4 (Monteiro and Feng, 2017). We noticed that the Venus-Shank3 band was indeed a double band, which may be the result of alternative splicing. To complete the characterization of endogenous Venus-Shank3 expression, immunocytochemistry on hippocampal neuronal cultures from Shank3^Venus/Venus^ mice shows that labeled endogenous Venus-Shank3 proteins are expressed in a punctate manner along dendrites (Figure 1D), as expected from the endogenous expression of Shank3 in dendritic spines (Tu et al., 1999).

### Shank3^Venus/Venus^ KI mice exhibit typical development and behavior

**FIGURE 2:**
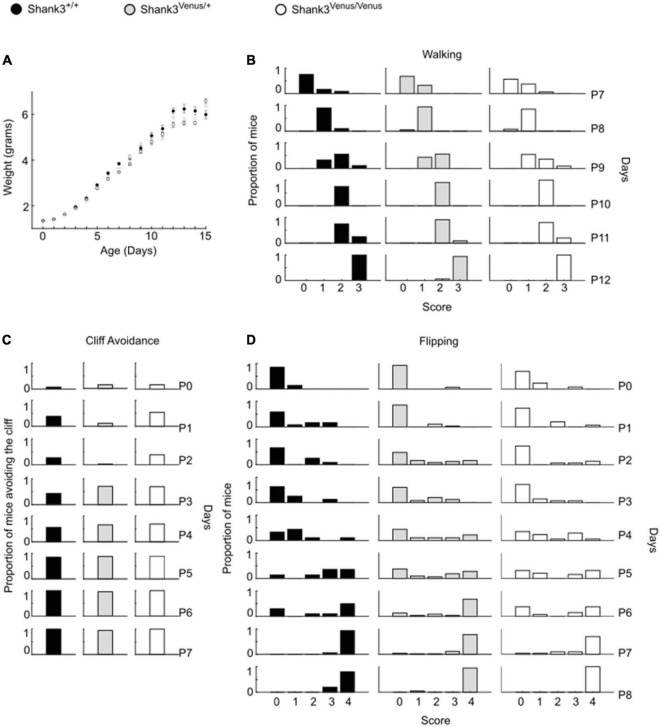
Psychomotor ability test battery in Shank3^Venus/Venus^, Shank3^Venus/+^ and Shank3^+/+^ KI pups. All behavioral experiments were performed on at least 18 mice per group. **(A)** *Weight.* Pups were weighed from birth to P15. Data are mean ± SEM. **(B)** *Walking.* Mice were scored for walking from P7 to P12. Data are proportion of mice reaching the different score through days. GLM, genotype effect: *p*-value = 0.81, interaction genotype by time: *p*-value = 0.48. **(C)** *Cliff Avoidance.* Proportion of mice avoiding the cliff from birth to P7. GLM, *p*-value = 1.00. **(D)** *Flipping*. Mice were scored in their ability to flip over when put on their back from birth to P8. Data are proportion of mice reaching the different score through days. GLM, *p*-value = 0.64.

**FIGURE 3:**
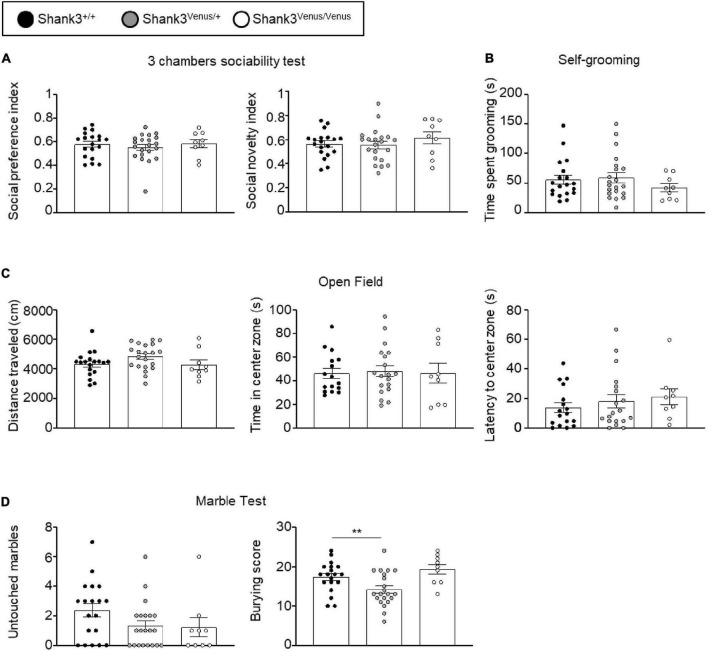
Behavioral test battery in Shank3^Venus/Venus^, Shank3^Venus/+^, and Shank3^+/+^ KI adult mice. **(A)** Three chambers sociability test. Social preference index: time spent in social stimulus side chamber divided by time spent in both chambers. Social novelty index: time spent in the novel social stimulus side chamber divided by time spent in both chambers. One-way ANOVA, *p*-values = 0.71 and 0.51 for social preference and social novelty indexes respectively. **(B)** Self-grooming. Time spent by the mice grooming during a 10 min period. Kruskal–Wallis test, *p*-value = 0.48. **(C)** Open Field. Total distance traveled by the mice during the 10 min test. One-way ANOVA, *p*-values = 0.13. Time spent in the center zone. One-way ANOVA, *p*-values = 0.95; latency to the first entry into the center zone. Kruskal–Wallis, *p*-value = 0.38. **(D)** Marble burying. Number of marbles untouched. Kruskal–Wallis, *p*-value = 0.11. Score of marble burying. One-Way ANOVA, *p*-value = 0.004. Bars are mean ± SEM. from 19 Shank3^+/+^, 21 Shank3^Venus/+^, and 9 Shank3^Venus/Venus^ except for Open field’s time spent in the center zone and latency to the center zone where data are from 17 Shank3^+/+^, 19 Shank3^Venus/+^, and 9 Shank3^Venus/Venus^.

We evaluated Shank3^Venus/+^ and Shank3^Venus/Venus^ mice metabolic and sensorimotor development by tracking mice weight, sensorimotor reflexes (flipping and cliff avoidance tests), and walking abilities (Figure 2). We used mice from postnatal day 0 (P0) to 15 (P15) to detect possible early deficits. We found no significant difference in weight from birth until P15 between Shank3^Venus/+^ and Shank3^+/+^ littermates. Similarly, flipping, cliff avoidance, and walking scores were not significantly affected in Shank3^Venus/+^ mice compared to WT mice. Because Shank3a mutations are associated with ASD like phenotypes, to rule-out any behavioral effect of the fusion of the Venus tag on Shank3a, we further characterized 12–14 weeks adult mice behaviors in tests known to reveal autistic-like phenotypes at this age. We evaluated mice social interests using the three chambers test. We found no difference between Shank3^+/+^, Shank3^Venus/+^, and Shank3^Venus/Venus^ mice in social preference nor in social novelty, highlighting the absence of atypical social interest in these KI mice (Figure 3A). We assessed stereotyped behaviors and anxiety measuring self-grooming, locomotion in the open field and mice propensity to interact with marbles. The time spent grooming was not significantly different between Shank3^+/+^, Shank3^Venus/+^, and Shank3^Venus/Venus^ mice (Figure 3B), suggesting the absence of stereotyped behavior and no atypical anxiety level in Venus-tagged Shank3a mice. The time spent in the center zone of an open field (Figure 3C) was not significantly different between genotypes either, suggesting the absence of atypical anxiety. We however noticed a slight but statistically significant decrease in burying score of Shank3^Venus/+^ heterozygous mice compared to Shank3^+/+^ in the marble test (Figure 3D) meaning that this genotype tends to burry more marbles. Yet, the propensity to interact with the marbles (number of untouched marbles) remained the same. As Marble burying test can be used as a proxy to measure anxiety, the outcome of this test reflects complex behavior combining anxiety together with novelty avoidance, stereotypical movements and general locomotion. That is why we performed supplemental behavioral tests for this genotype: Elevated Plus Maze test confirmed that anxiety-like behaviors of Shank3^Venus/+^ adult mice were typical (Supplementary Figure 1A). Performance in the Cyclotron (Supplementary Figure 1B) and Rotarod (Supplementary Figure 1C) showed that motor activity, motor coordination and endurance were typical as well. Aforementioned absence of hyper self-grooming ruled out stereotypical movements’ abnormalities. Altogether, these behavioral experiments show that Shank3^Venus/+^ and Shank3^Venus/Venus^ KI mice behave as WT mice. In future studies, Shank3^Venus/Venus^ KI mice can then be crossed with Shank3 mutant mouse models of ASD to understand the consequences of a Shank3 ASD mutation on the molecular dynamics of Venus-Shank3a at synapses in heterozygous mice.

### Shank3^Venus/Venus^ KI mice exhibit typical expression of Shank3 isoforms in the brain

**FIGURE 4:**
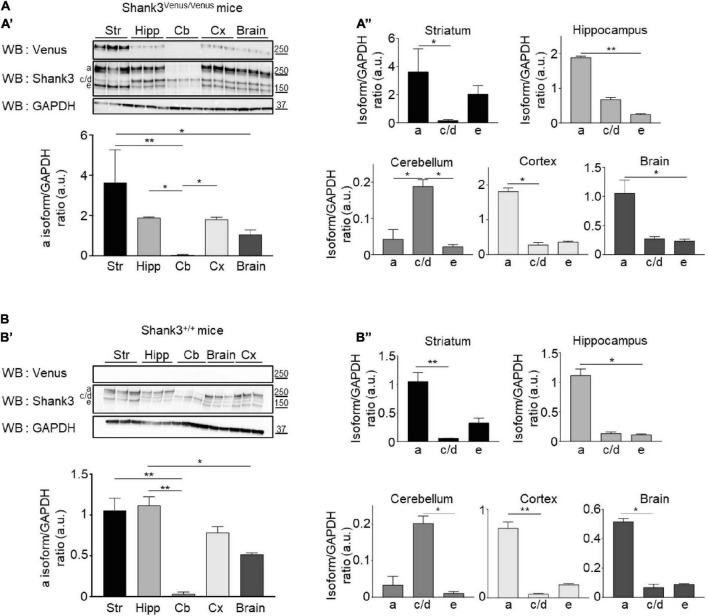
Shank3 protein isoforms expression in different brain areas. (**A**, Top **A’**) Total lysates of striatum (Str), hippocampus (Hipp), cerebellum (Cb), cortex (Cx), and full brain from 3 different Shank3^Venus/Venus^ adult mice immunoblotted for Venus, Shank3 and GAPDH. (Bottom **A’**) Summary graph of the Shank3a/GAPDH ratios (quantified using Shank3 antibody). (**A”**) Graphs present the Shank3a, Shank3c/d and Shank3e over GAPDH ratios in the full brain or the different brain areas (quantified with Shank3 antibody). **(B)** Same as panel **(A)** but in 3 Shank3^+/+^ adult mice. For panels **(A,B)** data are mean ± SEM, *indicates *p*-value < 0.05, **p < 0.01, non-parametric Kruskal–Wallis test with uncorrected Dunn’s post-test.

To compare the expression of the different Shank3 isoforms in Shank3^Venus/Venus^ (Figure 4A) and wild type mice (Figure 4B), Western blotting analysis was conducted on adult brain samples using Shank3 antibody. The Shank3a isoform is highly expressed in the striatum, hippocampus and cortex (Figures 4A’,B’) while the c/d isoforms predominate in the cerebellum (Figures 4A”,B”). Shank3e is present but weakly expressed in all brain areas studied. Relative to the other variants, it is most highly expressed in the striatum. We did not find major differences in Shank3 variants expression profile between Shank3^Venus/Venus^ mice and Shank3^+/+^ littermates. The absence of signal using the anti-Venus antibody on Western blot from Shank3^+/+^ mouse brain extracts (Figure 4B), which does not express Venus-tagged proteins, confirmed the specificity of endogenous Venus-Shank3a isoform detection by Venus tagging.

**FIGURE 5:**
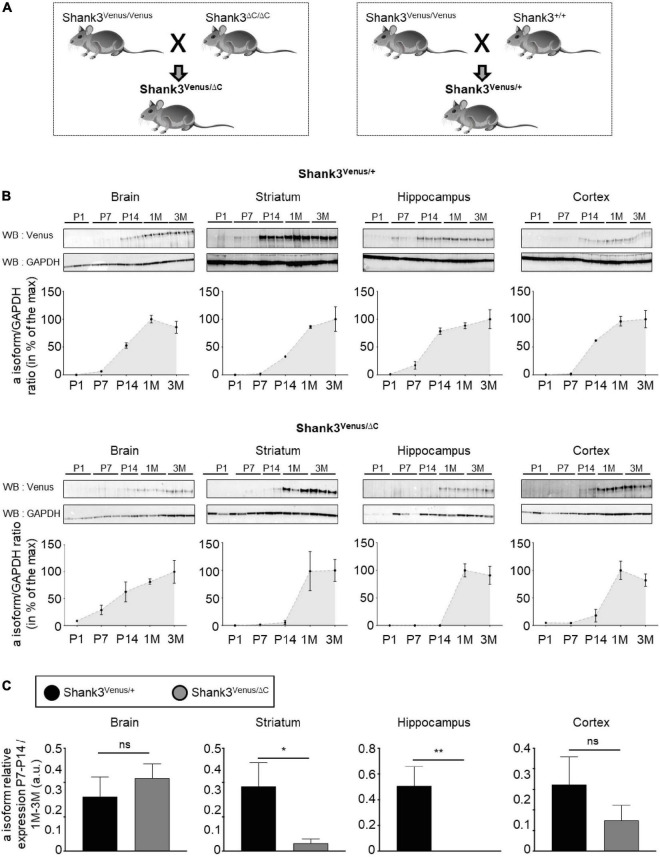
Expression of Shank3a isoform in ASD. **(A)** Mouse lines crossing scheme. **(B)** Total lysates extracted from full brain, striatum, hippocampus, and cortex of Shank3^Venus/+^ (top) or Shank3^Venus/ΔC^ (bottom) mice at different developmental stages, from Postnatal day 1 (P1) to 3 months-old (3M), immunoblotted for Venus and GAPDH. All replicates are presented, each from a different mouse. Data are expressed as a percentage of the maximum Shank3a/GAPDH ratio average. **(C)** Shank3a/GAPDH ratio in juvenile (P7 and P14) mice, expressed as a percentage of expression in adult mice (1 and 3 months) quantified in different brain regions from Shank3^Venus/+^ (black) and Shank3^Venus/ΔC^ (gray) mice. *n* = 6 samples from 6 different mice for each structure at each time point, excepted for Shank3^Venus/ΔC^ mice at P7–14 where *n* = 5. Data are mean ± SEM. ns: not significant; *p*-value: *<0.05; *p*-value: **<0.01 Mann–Whitney test.

Overall, these experiments using Shank3^Venus/Venus^ mice confirm a predominant expression of Shank3a in the striatum, hippocampus and cortex.

### Crossing Shank3^Venus/Venus^ KI mice with the Shank3^ΔC/ΔC^ mouse model of ASD reveals altered expression of the wild type Shank3a isoform in ASD

The Shank3ΔC mouse line displays a mutation found in humans and is commonly used to investigate autistic-like phenotypes (Kouser et al., 2013; Duffney et al., 2015; Moutin et al., 2021). We crossed *Shank3^Venus/Venus^* mice with *Shank3*^Δ*C/*Δ*C*^ or *Shank3*^+/+^ mouse strains to compare heterozygous mice expressing either Venus-Shank3 and untagged wild type Shank3 (*Shank3^Venus/+^*) or Venus-Shank3 and untagged Shank3ΔC (*Shank3*^*Venus/*Δ*C*^), Figure 5A. This allowed us to study Venus-Shank3a isoform expression in the context of ASD. In *Shank3^Venus/+^* mice, Venus-Shank3a was poorly expressed until postnatal day 7 in all studied brain regions (Figure 5B). At postnatal day 14, it considerably increased and reached a plateau at one month. These Venus-Shank3a expression kinetics were modified in *Shank3^Venus/ΔC^* mice. Indeed, the expression of Venus-Shank3a appeared to be delayed in the striatum and hippocampus, until one month. To better characterize this developmental gap in Venus-Shank3a expression, we directly compared Venus-Shank3a expression between the two genotypes at the critical period of synaptogenesis in juvenile (P7–14) mice (Figure 5C). Venus-Shank3a expression in the juvenile group (P7–14) was normalized by Shank3a expression in the adult group (1–3M), in both *Shank3^Venus/+^* and *Shank3*^*Venus/*Δ*C*^ genotypes. We confirmed a significant decrease of Venus-Shank3a expression in juvenile mice when co-expressed with ShankΔ3C, in the striatal and hippocampal regions. This alteration was absent when analyzing the full brain and only a tendency in the cortex. Altogether, these experiments show that the *Shank3^Venus/Venus^* KI mouse line can be crossed with ASD Shank3-mutant mice to track the expression of Venus-Shank3a isoform. The first results obtained with *Shank3^Venus/Venus^* KI mouse line revealed a delay in Venus-Shank3a expression during the postnatal critical period of development (P14) in the *Shank3ΔC* mouse model of ASD.

## Discussion

Shank3 is a key scaffolding protein that organizes glutamatergic postsynapse architecture and function (Boeckers et al., 2002; Monteiro and Feng, 2017). Many Shank3 isoforms are expressed in the brain, each one has its own unique specificity of brain area, cell type or even subcellular expression and each interacts with a specific plethora of proteins, strongly suggesting differential functions for each isoform (Wang et al., 2014; Monteiro and Feng, 2017). The longest and major Shank3 isoform has never been selectively studied due to the lack of specific antibodies or genetic tools enabling to isolate its functions from that of other isoforms. Here, we developed a transgenic *Shank3^Venus/Venus^* KI mouse to specifically study this Shank3a isoform, in its intact cellular context, at endogenous expression levels. Moreover, as a proof of concept, we show that this mouse line can be crossed with Shank3 mutant mice to study the Venus-Shank3a wild type protein in heterozygous mice models of ASD.

Although studies with overexpressed proteins have been essential so far to highlight different Shank isoforms specific functions (Han et al., 2013; Eltokhi et al., 2021), genome editing is a powerful technique for studying gene expression and functions in an intact cellular environment. The main advantage of this strategy precisely resides in its non-invasive nature, which circumvents protein overexpression frequently resulting from the use of mammalian-cell gene delivery vectors, such as viruses. Hence, tagging the endogenous protein minimizes experimental bias which may arise from aberrant protein expression or from deficient protein-protein interactions when the stoichiometry of functional complexes is altered. In agreement with these considerations, we found that the endogenous Venus-Shank3a protein in the KI mice displays similar developmental expression profile to the untagged endogenous Shank3a (Wang et al., 2014; Monteiro and Feng, 2017), it is correctly targeted to dendritic spines, the overall metabolic and sensorimotor development of the *Shank3^Venus/Venus^* KI mice is typical and adult *Shank3^Venus/Venus^* KI mice do not display autistic-like features. On the other hand, respecting endogenous expression levels can complicate the performance and analysis of experiments that rely on the fluorescence of a small number of Venus-tagged proteins at the single cell level. We by-passed the problem of weak fluorescence by amplifying the Venus fluorescence using fluorescent antibodies raised against Venus. Overall, we showed that the *Shank3^Venus/Venus^* KI mouse line provides a potential tool to elucidate the localization of Shank3a endogenous proteins in neurons by Venus tagging without altering Shank3 isoforms expression and preserving typical development and behavior of the mice. The morphology of *Shank3^Venus/Venus^* hippocampal neurons in culture is spiny, with a punctiform distribution of Venus-Shank3 at synapses. These mice will represent a powerful tool, by circumventing the lack of isoform specific antibodies, to investigate molecular dysfunctions at the synaptic level in various shankopathies (by crossing this line with mice carrying Shank3 mutations).

In a first set of experiments, Western blot analysis of the transgene expression revealed that only the Shank3a isoform was tagged with Venus. This observation is coherent with the genome editing chosen strategy, which was to add the coding sequence of Venus in frame with Shank3 exon1. Shank3 exon1 is absent from Shank3 b to f isoforms, each of them being produced by gene transcription starting at specific promoters located downstream of exon2. Hence as expected, only the isoform driven by the first promoter (upstream exon1) could display the Venus tag, i.e., the Venus-Shank3a isoform. However, downstream from the first transcription start site, *SHANK3* gene has alternative options of splicing, which could have resulted in the generation of a wide array of mRNA transcripts and protein isoforms. Alternative splicing of exon (exon 11, exon 12, exon 18, exon 21, and exon 22) have indeed already been shown to trigger the variety of additional transcripts for Shank3c, 3d, and 3e (Jiang and Ehlers, 2013). Our biochemical data revealed a unique band for Venus-Shank3 (Figure 1C), the Shank3a full-length, which appears, however, as a doublet. Hence, even if no alternative Shank3a isoform has been reported for so far (Monteiro and Feng, 2017), this doublet could indeed arise from alternative splicing. Alternatively, post-translational modifications, such as Shank3a phosphorylation (Wu et al., 2022), would similarly induce a slight shift in molecular weight. Future experiments may test these hypotheses, which are not mutually exclusive.

Consistent with previous reports (Lim et al., 1999; Wang et al., 2014; Lee et al., 2015; Mei et al., 2016), we found that the different Shank3 protein isoforms are differently expressed according to developmental stages and in a brain region specific manner, suggesting the existence of isoform specific functions. Systematic analysis of isoform-specific binding partners and isoform-specific brain expression patterns is an important step for future research. In that attempt, we used the herein generated Shank3^Venus/Venus^ KI mouse line in which endogenous Shank3a full-length isoform is specifically tagged to follow its neurodevelopmental expression. Our data show that Venus-Shank3a is mainly expressed in the cortex, striatum and hippocampus at early developmental stage when synaptogenesis occurs (P14). This specific brain area expression persists in the adult brain with an additional delayed expression in the adult cortex. We found no expression of Venus-Shank3a in the cerebellum, or a marginal one.

Given the potential importance of the *SHANK3* gene in ASD (Betancur and Buxbaum, 2013; Carbonetto, 2013; Leblond et al., 2014; Sala et al., 2015), it is crucial to understand more about its physiological role at the synapse and how it is disrupted by mutations. In humans, *SHANK3* gene (22q13.3) deletion triggers neurodevelopmental disorders like Phelan–McDermid syndrome (PMS), characterized by autistic like behaviors, hypotonia and delayed or absent speech (Bonaglia et al., 2001; Phelan et al., 2001; Phelan, 2008; Phelan and McDermid, 2012). Shank3 protein truncation is thought to cause the core neurodevelopmental and behavioral deficits that are observed in patients. By definition, in this heterozygous condition only one of the two Shank3 alleles displays the mutation. This means that the core PMS symptoms could either be triggered by Shank3 haploinsufficiency (Phelan and McDermid, 2012), and/or by a dominant negative effect of the Shank3 mutant form (resulting from aberrant trafficking to the dendritic spines or lack of interaction with functional partners). The latter hypothesis is more frequently investigated (Yoo et al., 2013; Sala et al., 2015; Varghese et al., 2017). Our strategy, focusing on the expression and role of the non-mutated endogenous Venus-Shank3a when co-expressed with the truncated untagged form, Shank3ΔC, complements the previous attempts to understand how alteration of the Shank3 protein can lead to the development of ASD. Here we showed that Venus-Shank3a expression is delayed during brain development when co-expressed with Shank3ΔC. In particular, Venus-Shank3a expression is deficient around P7-14, a period during which Shank3 is essential because of its role in the synaptogenesis and neuronal wiring of the developing brain (Sala et al., 2001, 2015; Roussignol et al., 2005; Durand et al., 2012; Macgillavry et al., 2016; Sarowar and Grabrucker, 2016; Pagani et al., 2019). We observed this delayed expression in the cortex, striatum and hippocampus. Defect in neuronal connectivity within the limbic system could explain, at least in part, the cognitive deficits associated with ASD. Hence, ASD may also depend on the ability of the wild type Shank3 allele to be typically expressed and engaged into functional complexes at synapses. Similarly, the difference in impairment severity might be explained not only by the expression pattern of Shank3 mutated form but also by the extent to which the spared Shank3 protein can (or cannot) compensate for its loss.

To conclude, the Shank3^Venus/Venus^ KI mouse line enables to track the Shank3a isoform in physiological and ASD conditions. This mouse line will be further useful to screen for differential Venus-Shank3a protein interactors in physiological and pathological conditions. Understanding Shank3 proteins function from an isoform specific perspective may also help to explain how different *SHANK3* gene mutations may result in distinct phenotypic consequences in different transgenic mice (Monteiro, 2018). Importantly, isoform-specific studies will help understanding the clinical conditions of patients with SHANK3 mutations, for whom genotype–phenotype stratification will help the design of specific pharmacological agents.

## Data availability statement

The original contributions presented in this study are included in the article/Supplementary material, further inquiries can be directed to the corresponding authors.

## Ethics statement

The animal study was reviewed and approved by the French Ministry of Research (APAFIS#23357-2019112715218160 v4 APAFIS#23476-2020010613546503 v4). Written informed consent was obtained from the owners for the participation of their animals in this study.

## Author contributions

EM and JP conceived research and wrote the manuscript. JP and FR designed the genome editing strategy. NB, SS, FR, FB, JP, and EM designed research experiments. NB, SS, and EM performed mice genotyping. VC and EM performed immunocytochemistry. A-LH-G performed tissue dissections. SS, EM, and FB performed behavioral experiments. VS, NB, and EM performed Western blots. JP supervised the project. All authors contributed to the preparation of the manuscript and approved it.

## References

[B1] American Psychiatric Association (2013). *Diagnostic and statistical manual of mental disorders (DSM-5)*, 5th Edn. Washington, DC: American Psychiatric Association. 10.1176/appi.books.9780890425596

[B2] Betancur C. Buxbaum J. D. (2013). SHANK3 haploinsufficiency: A “common” but underdiagnosed highly penetrant monogenic cause of autism spectrum disorders. *Mol. Autism* 4:17. 10.1186/2040-2392-4-17 23758743 PMC3695795

[B3] Boeckers T. M. Bockmann J. Kreutz M. R. Gundelfinger E. D. (2002). ProSAP/Shank proteins – A family of higher order organizing molecules of the postsynaptic density with an emerging role in human neurological disease. *J. Neurochem.* 81 903–910. 10.1046/j.1471-4159.2002.00931.x 12065602

[B4] Bonaglia M. C. Giorda R. Borgatti R. Felisari G. Gagliardi C. Selicorni A. (2001). Disruption of the ProSAP2 gene in a t(12;22)(q24.1;q13.3) is associated with the 22q13.3 deletion syndrome. *Am. J. Hum. Genet.* 69 261–268. 10.1086/321293 11431708 PMC1235301

[B5] Bourgeron T. (2015). From the genetic architecture to synaptic plasticity in autism spectrum disorder. *Nat. Neurosci.* 16 551–563. 10.1038/nrn3992 26289574

[B6] Brown E. A. Lautz J. D. Davis T. R. Gniffke E. P. Vanschoiack A. A. W. Neier S. C. (2018). Clustering the autisms using glutamate synapse protein interaction networks from cortical and hippocampal tissue of seven mouse models. *Mol. Autism* 9:48. 10.1186/s13229-018-0229-1 30237867 PMC6139139

[B7] Carbonetto S. (2013). A blueprint for research on shankopathies: A view from research on autism spectrum disorder. *Dev. Neurobiol.* 74 85–112. 10.1002/dneu.22150 24218108

[B8] Connor E. C. O. Bariselli S. Bellone C. (2014). Synaptic basis of social dysfunction: A focus on postsynaptic proteins linking group- I mGluRs with AMPARs and NMDARs. *Eur. J. Neurosci.* 39 1114–1129. 10.1111/ejn.12510 24712991

[B9] Costales J. L. Kolevzon A. (2015). Phelan – McDermid syndrome and SHANK3: Implications for treatment. *Neurotherapeutics* 12 620–630. 10.1007/s13311-015-0352-z 25894671 PMC4489957

[B10] Duffney L. J. Zhong P. Buxbaum J. D. Yan Z. Duffney L. J. Zhong P. (2015). Autism-like deficits in Shank3-deficient mice are rescued by targeting actin regulators. *Cell Rep.* 11 1400–1413. 10.1016/j.celrep.2015.04.064 26027926 PMC4464902

[B11] Durand C. M. Betancur C. Boeckers T. M. Bockmann J. Chaste P. Fauchereau F. (2007). Mutations in the gene encoding the synaptic scaffolding protein SHANK3 are associated with autism spectrum disorders. *Nat. Genet.* 39 25–27. 10.1038/ng1933 17173049 PMC2082049

[B12] Durand C. Perroy J. Loll F. Perrais D. Fagni L. Bourgeron T. (2012). SHANK3 mutations identified in autism lead to modification of dendritic spine morphology via an actin-dependent mechanism. *Mol. Psychiatry* 1757 71–84. 10.1038/mp.2011.57 21606927 PMC3252613

[B13] Eltokhi A. Gonzalez-Lozano M. A. Oettl L. L. Rozov A. Pitzer C. Röth R. (2021). Imbalanced post- and extrasynaptic SHANK2A functions during development affect social behavior in SHANK2-mediated neuropsychiatric disorders. *Mol. Psychiatry* 26:6505. 10.1038/s41380-021-01140-y 34234282 PMC9119257

[B14] Han K. Holder J. L. Schaaf C. P. Lu H. Chen H. Kang H. (2013). SHANK3 overexpression causes manic-like behaviour with unique pharmacogenetic properties. *Nature* 503 72–77. 10.1038/nature12630 24153177 PMC3923348

[B15] Jiang Y. Ehlers M. D. (2013). Modeling autism by SHANK Gene mutations in mice. *Neuron* 78 8–27. 10.1016/j.neuron.2013.03.016 23583105 PMC3659167

[B16] Kleijer K. T. E. Schmeisser M. J. Bourgeron T. Brose N. Burbach J. P. H. (2014). Neurobiology of autism gene products: Towards pathogenesis and drug targets. *Psychopharmacology (Berl)* 231 1037–1062. 10.1007/s00213-013-3403-3 24419271

[B17] Kouser M. Speed H. E. Dewey C. M. Reimers J. M. Widman A. J. Gupta N. (2013). Loss of predominant Shank3 isoforms results in hippocampus-dependent impairments in behavior and synaptic transmission. *J. Neurosci.* 33 18448–18468. 10.1523/JNEUROSCI.3017-13.2013 24259569 PMC3834052

[B18] Leblond C. S. Nava C. Polge A. Gauthier J. Huguet G. Lumbroso S. (2014). Meta-analysis of SHANK mutations in autism spectrum disorders : A gradient of severity in cognitive impairments. *PLoS Genet.* 10:e1004580. 10.1371/journal.pgen.1004580 25188300 PMC4154644

[B19] Lee E. Lee J. Kim E. (2016). Review excitation / inhibition imbalance in animal models of autism spectrum disorders. *Biol. Psychiatry* 81 838–847. 10.1016/j.biopsych.2016.05.011 27450033

[B20] Lee J. Chung C. Ha S. Lee D. Kim D. Y. Kim H. (2015). Shank3-mutant mice lacking exon 9 show altered excitation/inhibition balance, enhanced rearing, and spatial memory deficit. *Front. Cell Neurosci.* 9:94. 10.3389/fncel.2015.00094 25852484 PMC4365696

[B21] Lim S. Naisbitt S. Yoon J. Hwang J. I. Suh P. G. Sheng M. (1999). Characterization of the Shank family of synaptic proteins. Multiple genes, alternative splicing, and differential expression in brain and development. *J. Biol. Chem.* 274 29510–29518. 10.1074/jbc.274.41.29510 10506216

[B22] Macgillavry H. D. Kerr J. M. Kassner J. Frost N. A. Blanpied T. A. (2016). Shank – cortactin interactions control actin dynamics to maintain flexibility of neuronal spines and synapses. *Eur. J. Neurosci.* 43 179–193. 10.1111/ejn.13129 26547831 PMC5007541

[B23] Mei Y. Monteiro P. Zhou Y. Kim J.-A. Gao X. Fu Z. (2016). Adult restoration of Shank3 expression rescues selective autistic-like phenotypes. *Nature* 530 481–484. 10.1038/nature16971 26886798 PMC4898763

[B24] Monteiro P. (2018). Shank3 mutations and HCN channelopathy: One size does not fit all. *J. Physiol.* 596:1123. 10.1113/JP275828 29427308 PMC5878215

[B25] Monteiro P. Feng G. (2017). SHANK proteins: Roles at the synapse and in autism spectrum disorder. *Nat. Rev. Neurosci.* 18 147–157. 10.1038/nrn.2016.183 28179641

[B26] Moutin E. Hemonnot A.-L. Seube V. Linck N. Rassendren F. Perroy J. (2020). Procedures for culturing and genetically manipulating murine hippocampal postnatal neurons. *Front. Synaptic Neurosci.* 12:19. 10.3389/fnsyn.2020.00019 32425766 PMC7204911

[B27] Moutin E. Sakkaki S. Compan V. Bouquier N. Giona F. Areias J. (2021). Restoring glutamate receptosome dynamics at synapses rescues autism-like deficits in Shank3-deficient mice. *Mol. Psychiatry* 26 7596–7609. 10.1038/s41380-021-01230-x 34331007

[B28] Naisbitt S. Kim E. Tu J. C. Xiao B. Sala C. Valtschanoff J. (1999). Shank, a Novel family of postsynaptic density proteins that binds to the NMDA Receptor / PSD-95 / GKAP complex and cortactin. *Neuron* 23 569–582. 10.1016/s0896-6273(00)80809-0 10433268

[B29] Pagani M. Bertero A. Liska A. Galbusera A. Sabbioni M. Barsotti N. (2019). Deletion of autism risk gene Shank3 disrupts prefrontal connectivity functional. *J. Neurosci.* 39 5299–5310. 10.1523/JNEUROSCI.2529-18.2019 31061091 PMC6607754

[B30] Phelan K. McDermid H. E. (2012). The 22q13.3 deletion syndrome (Phelan-McDermid syndrome). *Mol. Syndromol.* 2 186–201. 10.1159/000334260 22670140 PMC3366702

[B31] Phelan M. C. (2008). Deletion 22q13.3 syndrome. *Orphanet J. Rare Dis.* 3:14. 10.1186/1750-1172-3-14 18505557 PMC2427010

[B32] Phelan M. C. Curtis Rogers R. Saul R. A. Stapleton G. A. Sweet K. McDermid H. (2001). 22q13 deletion syndrome. *Am. J. Med. Genet.* 101 91–99. 11391650 10.1002/1096-8628(20010615)101:2<91::aid-ajmg1340>3.0.co;2-c

[B33] Roussignol G. Ango F. Romorini S. Tu J. C. Sala C. Worley P. F. (2005). Shank expression is sufficient to induce functional dendritic spine synapses in aspiny neurons. *J. Neurosci.* 25 3560–3570. 10.1523/JNEUROSCI.4354-04.2005 15814786 PMC6725374

[B34] Sala C. Piëch V. Wilson N. R. Passafaro M. Liu G. Sheng M. (2001). Regulation of dendritic spine morphology and synaptic function by Shank and Homer. *Neuron* 31 115–130. 10.1016/S0896-6273(01)00339-7 11498055

[B35] Sala C. Vicidomini C. Bigi I. Mossa A. Verpelli C. (2015). Shank synaptic scaffold proteins: Keys to understanding the pathogenesis of autism and other synaptic disorders. *J. Neurochem.* 135 849–858. 10.1111/jnc.13232 26338675

[B36] Sarowar T. Grabrucker A. M. (2016). Actin-dependent alterations of dendritic spine morphology in shankopathies. *Neural Plast.* 2016:8051861. 10.1155/2016/8051861 27795858 PMC5067329

[B37] Tu J. C. Xiao B. Naisbitt S. Yuan J. P. Petralia R. S. Brakeman P. (1999). Coupling of mGluR/Homer and PSD-95 complexes by the Shank family of postsynaptic density proteins. *Neuron* 23 583–592. 10.1016/S0896-6273(00)80810-7 10433269

[B38] Varghese M. Keshav N. Jacot-Descombes S. Warda T. Wicinski B. Dickstein D. L. (2017). Autism spectrum disorder: Neuropathology and animal models. *Acta Neuropathol.* 134 537–566. 10.1007/s00401-017-1736-4 28584888 PMC5693718

[B39] Verpelli C. Dvoretskova E. Vicidomini C. Rossi F. Chiappalone M. Schoen M. (2011). Importance of Shank3 protein in regulating metabotropic glutamate receptor 5 (mGluR5) expression and signaling at synapses. *J. Biol. Chem.* 286 34839–34850. 10.1074/jbc.M111.258384 21795692 PMC3186429

[B40] Wang X. Xu Q. Bey A. L. Lee Y. Jiang Y.-H. (2014). Transcriptional and functional complexity of Shank3 provides a molecular framework to understand the phenotypic heterogeneity of SHANK3 causing autism and Shank3 mutant mice. *Mol. Autism* 5:30. 10.1186/2040-2392-5-30 25071925 PMC4113141

[B41] Wu C. H. Tatavarty V. Beltran P. M. J. Guerrero A. Keshishian H. Krug K. (2022). A bidirectional switch in the shank3 phosphorylation state biases synapses toward up or down scaling. *Elife* 11:e74277. 10.7554/eLife.74277 35471151 PMC9084893

[B42] Yoo J. Bakes J. Bradley C. Collingridge G. L. Kaang B. (2013). Shank mutant mice as an animal model of autism. *Philos. Trans. R. Soc Lond. B Biol. Sci.* 369:20130143. 10.1098/rstb.2013.0143 PMC3843875 24298145

