# Supplementary material for: The Shank3Venus/Venus knock in mouse enables isoform-specific functional studies of Shank3a
Source: Front Neurosci. 2022 Dec 8;16:1081010. doi: 10.3389/fnins.2022.1081010 (PMC9773256; doi:10.3389/fnins.2022.1081010)
Supplement: Supplementary file 1 [file Data_Sheet_1.pdf]

Supplementary methods

Elevated plus maze, Cyclotron and Rotarod experiments were performed on 8-9 week-old mice males and females.

Elevated Plus Maze: assessment of anxiety level was measured using an elevated plus maze (two opposing open arms and two opposing closed arms, 40 cm above the ground). The number of entries in the open/closed arm and the time spent in each type of arm were measured over a 10 min period.

Cyclotron: Locomotion was recorded using circular corridors (Cyclotron, Imetronic, France). Each circular corridor is equipped of 4 infrared sensors (one for every quadrant) at ground level for recording horizontal movement. Another detector is positioned at a height of 7.5 cm to follow the animal rearings. Horizontal activity is the number of quadrants that the mouse has crossed every 5 minutes, while vertical activity corresponds to the number of rearing movements in a 5-minute period. Mice locomotion was recorded for a total of 60 min.

Rotarod: The Rotarod test was used to analyse motor coordination in mice. The apparatus is composed of a 3-cm-diameter rotating rod elevated 16 cm above a platform (Ugo Basile Microprocessor Controlled Rota-Rod Treadmills, Italy). Three trials per day were performed for two consecutive days, after a first day of training. For each trial, adult mice were placed on the rod at 4 rpm. Once stable, the accelerating mode started to reach 40 rpm in 5 min. The latency to fall of the animals was automatically recorded. A 20 min interval was used between sessions to avoid fatigue.

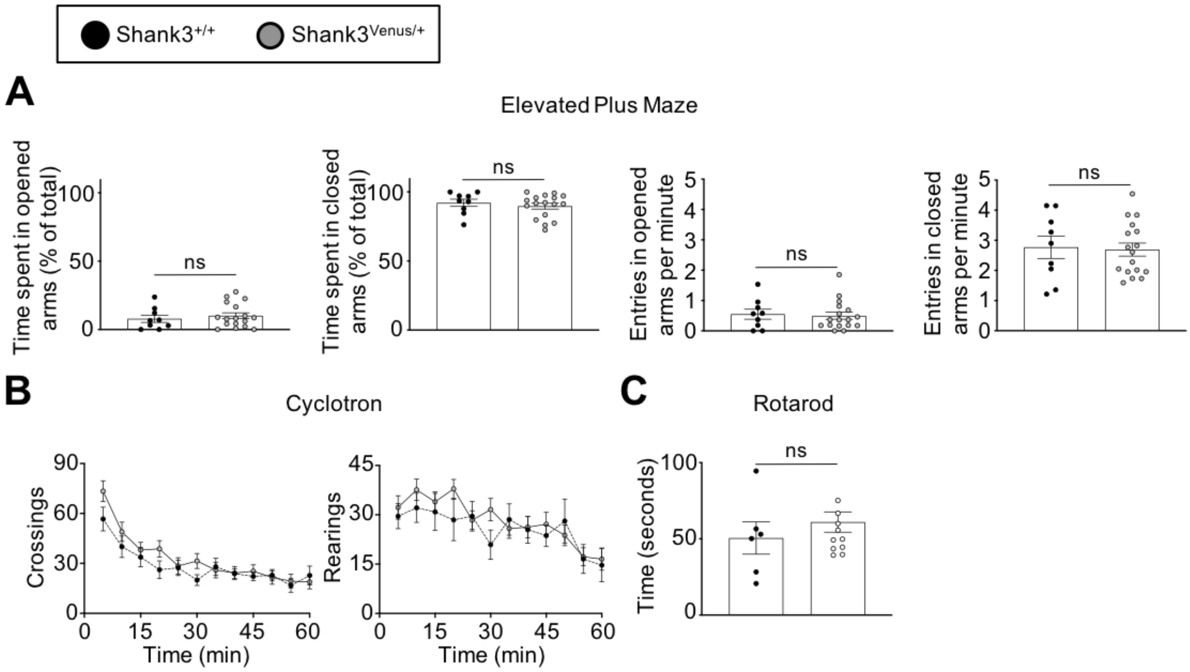

Supplemental Figure: Behavioral test battery in Shank3$^{Venus/+}$ adult mice. A: Elevated Plus Maze task. From left to right: Percentage of time spent in open arms; Percentage of time spent in closed arms; Number of entries in open arms per minute; Number of entries in closed arms per minute. Mann-Whitney test. Data are mean ± SEM from 9 Shank3$^{+/+}$ mice and 17 Shank3$^{Venus/+}$ mice. **B:** Cyclotron task. Left: number of crossings across time. Right, number of rearings. Data are mean ± SEM from 9 Shank3$^{+/+}$ mice and 17 Shank3$^{Venus/+}$ mice. Multiple unpaired t-test, FDR post-test with two-stage step-up method of Benjamini, Krieger and Yekutieli. **C:** Accelerating Rotarod. Averaged latency to fall off the rod during six consecutive sessions over two days. Mann-Whitney test. Data are mean ± SEM from 6 Shank3$^{+/+}$ mice and 12 Shank3$^{Venus/+}$ mice.
